# Does educational attainment matter for attitudes toward immigrants in Chile? Assessing the causality and generalizability of higher education's so‐called “liberalizing effect” on economic and cultural threat

**DOI:** 10.1111/1468-4446.13124

**Published:** 2024-06-21

**Authors:** Paolo Velásquez

**Affiliations:** ^1^ Stockholm School of Economics Stockholm Sweden; ^2^ Institute for Future Studies Stockholm Sweden

**Keywords:** attitudes, Chile, educational attainment, immigrants, Latin America, prejudice

## Abstract

Despite a large literature consistently showing a relationship between higher levels of education and lower levels of ethnic prejudice, some points of contention remain. First, it remains unclear whether education has a causal effect on attitudes, mainly due to a lack of longitudinal studies. Second, due to the majority of studies on prejudice being conducted in Europe and North America, we do not know to what extent the inverse relationship between education and prejudice is generalizable beyond the “global North.” To answer these questions, I study attitudes toward immigrants in Chile in the years 2016–2022, using six waves of the Chilean Longitudinal Social Survey. Chile provides new variations in economic and cultural factors, with its stable albeit highly unequal economy, and increased immigration from culturally similar countries which shed light on possible scope conditions of the so‐called liberalizing effect of education. I analyze whether *attaining* more education has an effect on reducing levels of perceived economic and cultural threat. The findings show that increases in education are associated with both lower levels of perceived economic and cultural threat, with education having a stronger effect on the latter.

## 1 INTRODUCTION

In studies of prejudice, research on native‐born attitudes toward ethnic minorities and immigrants has consistently found an inverse relationship between education and prejudice (e.g., Ceobanu & Escandell, 2010; Dražanová et al., 2023; Hainmueller & Hopkins, 2014; Pottie‐Sherman & Wilkes, 2017), making it, arguably, one of the most consistent findings in the social sciences (e.g., Hagendoorn & Nekuee, 1999; Vogt, 1997). This relationship has been shown to have a stronger effect in Western countries than in Eastern Europe (Coenders & Scheepers, 2003; Hjerm, 2001; Wagner & Zick, 1995), suggesting this relationship to be universal, although in varying degrees. In addition, this relationship is more salient when comparing individuals with and without tertiary education (Hainmueller & Hiscox, 2007; Velásquez & Eger, 2022). However, despite this robust result, a few points of contention remain.

First, the question of whether prejudice toward immigrants arises from perceptions of economic or cultural threats persists, especially in non‐Western contexts. This is mainly due to few studies on this topic, which raises further questions regarding education's potential to reduce prejudice. The literature on attitudes toward immigrants and immigration commonly distinguishes between economic or cultural threats, for example, immigration as a source of unemployment or dilution of national culture (Hjerm & Nagayoshi, 2011; Ivarsflaten, 2005; Lucassen & Lubbers, 2012; Schneider, 2008). Relatedly, studies also differentiate separate individual‐level determinants of anti‐immigrant attitudes, such as education and other socioeconomic indicators (e.g., income, occupation). In other words, immigration has been conceptualized as either an economic or a cultural issue at the societal level that matters for the native‐born and ethnic majority. Meanwhile, at the individual level, attitudinal determinants of anti‐immigrant sentiment diverge in respect to educational attainment on the one hand, and income and labor market position on the other (e.g., Hainmueller & Hiscox, 2007; Manevska & Achterberg, 2013), with the latter determinant of immigration attitudes receiving little support (Hainmueller & Hopkins, 2014; Jeannet, 2018).

Second, it remains somewhat unclear whether attaining more education decreases prejudiced attitudes or whether less‐prejudiced individuals self‐select into higher education (Lancee & Sarrasin, 2015; Velásquez & Eger, 2022; Weber, 2022). This gap in our understanding mainly stems from a lack of longitudinal studies, and the few studies that have analyzed this association over time have yielded different results. One possible explanation for these discrepancies may be due to the different ways anti‐immigrant sentiment is operationalized. On the one hand, studies that do not find any causal effect of education on prejudice have focused on equal opportunities for immigrants (Lancee & Sarrasin, 2015), feelings toward specific ethnic groups (Weber, 2022) and on the effect of field of study on an index of anti‐immigrant attitudes (Kunst, 2022). On the other hand, studies that do find support have focused on more general attitudes toward immigrants (Velásquez & Eger, 2022) and racial prejudice (Scott, 2022).

Third, it is unclear to what extent the relationship between education and prejudice is generalizable beyond the “global North.” Despite a great number of studies on anti‐immigrant sentiment, the vast majority have been conducted in Western Europe and North America. This is unfortunate for at least two reasons. The first reason is that at least 37% of the global migration flow takes place in the “global South” (IOM, 2017), that is, South‐South migration. This means that we lack substantial knowledge as whether the inverse relationship between education and prejudice manifests itself in other parts the world, and to what extent education matters for explaining anti‐immigrant sentiment. The second reason speaks to the universality of theories, and generalization of results in studies of anti‐immigrant sentiment and prejudice more broadly. Although results from the global North may be extrapolated to other parts of the world, we cannot be certain unless we anchor our theories to empirical evidence. For example, research has shown that people from Western, educated, industrialized, rich and democratic countries are rather a “thin slice of humanity” (Henrich et al., 2010), yet knowledge production in the global North is still assumed to be more universal than in the global South (Castro Torres & Alburez‐Gutierrez, 2022).

In an effort to help answer these important questions, I investigate to what extent education matters for attitudes toward immigrants in Chile. This case offers a good opportunity to study anti‐immigrant sentiment due to the country's recent history as an emerging high‐income economy accompanied by a rapid increase in immigration. Despite Chile being one the most stable economies in Latin America, it currently has the second highest level of inequality of any OECD country (OECD, 2022), and one of the highest in the world. In addition, the composition of the immigrant population is objectively similar to the native population, which largely shares the same language and religion. This combination of high economic inequality and objective cultural similarity to immigrants, makes Chile a compelling case for studying anti‐immigrant sentiment when contrasting it to the large number of studies conducted in Western countries that have considerably lower levels of inequality and more diverse immigrant ethnic compositions. This different context provides a new variation in economic and cultural factors which gives the opportunity to assess any possible scope conditions of the effect of education on attitudes toward immigrants.

The main research questions of this paper are: “does attaining more education reduce anti‐immigrant sentiment?” And if so, then “why might this be different in a country in the global South?” In order to answer these questions, I analyze six waves of the Chilean Longitudinal Social Survey (ELSOC), the first longitudinal study in Chile and Latin America. In the following sections, I present previous research defined in three themes: the link between education and prejudice, causality, and generalizability. First, I explain why education should theoretically matter for reducing prejudice and provide possible mechanisms responsible for this relationship. I then present previous research on whether education has a causal effect on attitudes. Lastly, I review previous studies in Chile and Latin America and motivate why the Chilean context is well‐suited to assess the effect of education on perceived economic and cultural threat.

## 2 PREVIOUS RESEARCH

### 2.1 The link between education and prejudice—Theoretical mechanisms

The literature on attitudes toward immigrants and ethnic/racial minorities often conceptualizes the effect of education in two ways: as a “socioeconomic effect” or a “liberalizing effect.” In the former, prejudice is seen as a response to perceived threat for economic resources (Blumer, 1958), with education representing human capital and one's ability to compete in the labor market. Education is thus considered an indicator of social position and treated as a class variable having “allocation effects” rather than having an independent effect or a “direct effect” (Stubager, 2008; Surridge, 2016). Prejudiced attitudes toward ethnic out‐groups consequently arise from the perceived threat experienced by individuals with fewer resources in direct competition with lower‐educated ethnic minorities, and this may be explained by “relative deprivation” (Runciman, 1972), where low‐status individuals feel they get less than what they deserve compared to others. Conversely, highly educated, high‐status individuals tend to be less prejudiced as they are economically secure and not in direct competition with low‐skilled immigrants. However, using education as an indicator of economic position faces challenges. Research indicates that education and other socioeconomic status (SES) variables yield distinct effects (e.g., Citrin et al., 1997; Hello et al., 2002). There is evidence that other SES indicators are not strongly associated with immigration attitudes (Hainmueller & Hopkins, 2014; Jeannet, 2018), challenging the idea that economic self‐interest is solely responsible. This suggests that higher education may be a proxy for a liberal orientation instead (Hello et al., 2002, 2006).

In regard to possible mechanisms responsible for the so‐called “liberalizing effect of education,” one mechanistic account posits that education has the potential to reduce prejudice by providing specific knowledge about civil liberties and fostering critical thinking skills (Nunn et al., 1978). Additional years of education have been shown to be associated with greater support for civil liberties (Ohlander et al., 2005), and with the development of critical thinking skills, by increasing awareness of diverse human experiences and the importance of extending civil liberties to all groups, even those we dislike (Bobo & Licari, 1989).

Second, higher levels of education may decrease prejudice by transmitting the dominant values in a society through the educational system (Phelan et al., 1995; Selznick & Steinberg, 1979). By spending more time in the educational system, exposure to these “official values” over time leads to internalization, whereas the uneducated reject these values and represent a divergent “common culture.” The formal educational system therefore promotes liberal values such as equality and freedom of expression. According to this view, the effect of education on prejudice is contingent on a country's history of liberal democracy, with prolonged and interrupted democracies having a stronger educational effect than recent democracies (Coenders & Scheepers, 2003; Dražanová, 2017).

Lastly, education may provide a sense of psychological security, with higher levels of education associated with better coping mechanisms for “deviant lifestyles” (Jenssen & Engesbak, 1994). Individuals with low levels of education may lack this security and exhibit weaker support for civil liberties (McClosky & Brill, 1983). Education contributes to a sense of “mastering one's own life situation,” leading to psychological stability and positive self‐worth. Psychologically secure individuals are thus better equipped to handle “big events” (Velásquez & Eger, 2022) and may exhibit greater tolerance toward ethnic out‐groups.

In sum, education is seen as having a so‐called “liberalizing effect,” by conferring democratic values, critical thinking skills, specific knowledge, or psychological security (Stubager, 2008; Surridge, 2016). Education having a “direct effect” has received greater empirical support than education as an indicator of economic position (e.g., Fetzer, 2000; Ivarsflaten, 2005; Manevska & Achterberg, 2013), with the link between education and attitudes toward immigrants and immigration being likely driven by conflicting views on values and beliefs (Hainmueller & Hiscox, 2007; Schneider, 2008).

### 2.2 Causality

Research has shown, time and time again that individuals with higher levels of education, and particularly tertiary education, hold more positive attitudes toward immigrants and immigration, whether education is the main focus of the study or included as a control variable (e.g., Ceobanu & Escandell, 2010; Dražanová et al., 2023; Hjerm, 2001). Recent studies, however, have challenged the assumption that education has a *causal* effect on prejudice, mainly due to a lack of longitudinal studies.

In recent years, a few studies in different European countries have tackled this question using longitudinal data, which have yielded mixed results. Lancee and Sarrasin (2015) looked at opinions of native Swiss on equal opportunities between Swiss citizens and immigrants. They concluded that attaining more education did not influence these attitudes, hinting at self‐selection effects. Similarly, in a study of students' attitudes over an 8‐year period in Germany, Weber (2022) analyzed answers to “feeling thermometer” questions for different ethnic out‐groups and found no causal evidence of transitioning from secondary to tertiary education. In the Netherlands, Kunst (2022) looked at differences between fields of education on anti‐immigration attitudes using a scale of six items and found no clear and robust causal effect in anti‐immigration sentiment. In Norway, Velásquez and Eger (2022) found evidence of a causal effect of attaining more education, in responding to whether immigrants settling in the country were more of an advantage than a disadvantage. In Great Britain, Scott (2022) reported individuals graduating with a university degree to be less racially prejudiced, although the focus was not on immigrants.

While these studies arrive at different conclusions, they share one commonality; namely that their dependent variables refer to different aspects of immigration without distinguishing between the (perceived) effect of immigrants on *economic* and *cultural* terms. Although studies of attitudes toward immigrants often amalgamate these two into an index, for example, “perceived ethnic threat” (e.g., Manevska & Achterberg, 2013; Schneider, 2008), I argue that making this distinction is relevant in the Chilean context, since educational attainment may potentially be associated more strongly with either lower levels of perceived economic or perceived cultural threat. Given the linguistic and religious similarities of native and immigrant populations in Chile, and the country's stable economy coupled with high levels of inequality, it offers a fertile ground for furthering our understanding of the specific elements related to cultural identity and economic concerns that influence immigration attitudes. It is not only important to examine *if* attaining more education lowers prejudice but also whether it affects economic or cultural concerns differently.

### 2.3 Generalizability

The majority of studies on prejudice and attitudes toward out‐groups have been conducted mainly in Western Europe, North America and other advanced economies. Yet, it is not certain whether these results are generalizable to a country like Chile, which has only in recent years seen steady economic growth accompanied by an increase in immigration. For example, in a study of Latin American countries, Meseguer and Kemmerling (2018) looked at anti‐immigrant sentiment based on fears of labor‐market competition and found no support for natives' belief that immigrants take their jobs, but found support for natives' fear of higher taxation as a consequence of increased immigration. In addition, the authors found education to be negatively correlated with anti‐immigrant sentiment at the individual‐level. At the country‐level, the highest levels of anti‐immigrant sentiment were found in countries with a low presence of predominantly educated immigrants. In a study of support for more restrictive immigration laws, Lawrence (2011) reported personal economic satisfaction, ability to meet basic needs and higher education to be associated with less support for restrictive immigration laws in Latin America.

In Chile, research toward ethnic minorities and immigrants is scarce, and studies focusing on the effect of education on these attitudes is even less common. In one of the first studies of prejudice against minority groups in Chile, González (2005) looked at primary and secondary school students' level of prejudice toward different out‐groups, and found that Roma, the homeless and Peruvians were the groups more discriminated against.^1^ However, secondary school students reported less prejudice toward Roma and Peruvians than those in primary school, where low‐SES students had significantly less positive attitudes than students with higher‐SES. Correspondingly, Carvacho et al. (2013) found that greater levels of educational attainment among adults, but not income, had a positive effect on attitudes toward Peruvians and Argentinians, when asked questions regarding esteem, trust, and admiration. Sirlopú and Van Oudenhoven (2013) have also found Chileans of low SES to report less support for multiculturalism and more negative emotions and feelings of threat toward Peruvian immigrants. More recently, in a study of support for work visas, Lawrence (2015) showed that concerns over job competition are a poor predictor of immigration attitudes, suggesting that it is rather the broader economic implications to the country as a whole that are of importance. Further, the author found that highly educated individuals (engineers) did not feel threatened by immigrants pursuing work in their field.

The literature on anti‐immigrant sentiment in Chile and Latin America has mainly focused on economic concerns while largely neglecting cultural aspects. While immigration in Chile has not affected employment levels, it has been misperceived as increasing unemployment (Ajzenman et al., 2022). At first glance this is logical since Latin America is a highly unequal region and countries share similar cultures. However, even if countries in Latin America are similar in many aspects, there are still reasons to believe cultural threats play a role in anti‐immigrant sentiment.

Recent immigrants to Chile come mainly from other Latin American countries and can be considered *relatively* similar to Chileans. They share a common history as colonized countries, speak the same language (except for Haitians immigrants who speak French or Creole), and share the same religion. Research suggests that subjectively perceived dissimilarities may contribute to higher levels of perceived cultural threat among groups that are (objectively) culturally similar (Zárate et al., 2004). Indeed, attributes shared by ethnic groups that seem strikingly similar to outsiders may not actually matter much to members of either group. While two ethnic groups can be considered similar, members of these ethnic groups may emphasize irreconcilable differences. Little research has been done on attitudes toward culturally similar immigrants. One exception is Helbling (2011) who shows that the Swiss consider Germans less likeable than other Western Europeans, such as the French and Italians; and emphasizes the importance of looking at how out‐groups are conceived by in‐group members instead of relying on based on what appears to be objective criteria.

According to Theiler (2004), large cultural differences are perceived as unproblematic, whereas small cultural differences are usually fragile and can often lead to fears of inadequate separation from other groups. This may be the case in Chile, where despite apparent similarities, “there may be significant tensions … stemming from differences that are regarded as fundamental by one or both groups” (Helbling, 2011, p. 13). Indeed, Chileans' collective identity is characterized by a belief in a culturally and ethnic homogenous nation, highlighting their European ancestry and preferring fair‐skinned people (Bonhomme, 2023; González et al., 2016; Sirlopú & Van Oudenhoven, 2013; Uhlmann et al., 2002). In this manner, although there are objective similarities between Chileans and immigrants from other Latin American nations, they are likely to be subjectively perceived as essentially different and as a cultural threat to the national identity.

## 3 CHILEAN CONTEXT

In the last few decades, immigration to Chile has increased rapidly, mainly from neighboring countries and other Latin American nations. Historically, Chile has not been a popular migration destination compared to other countries in the Atlantic coast (Argentina, Brazil, and Uruguay). However, this began to change after the return of democracy in 1990 and an economic boom that has attracted many immigrants from other Latin American countries. In 2006, the immigrant population in Chile was a mere 0.96% of the population, while in 2017 it reached 4.5% (Fuentes & Hernando, 2020). More recent numbers estimate the immigrant population to be around 9% in 2022^2^ or 1,625,074 people (INE, 2023), making it the most immigrants Chile has had in its history since 1907 where 4.1% of the population was foreign born (Braun et al., 2000). The four largest immigrant groups as of 2022 were: Venezuelans (32.8%), Peruvians (15.3%), Colombians (11.7%), and Haitians (11.4%).

Chile has also enjoyed rising living standards in the last three decades, with one of the highest GDPs per capita in Latin America at $23,455 US dollars in 2021 (World Bank, 2022). Despite its economic progress, Chile remains one of the most unequal countries in the world, with the concentration of income held by the lowest 50% to be 2.1% and the richest 10% holding two‐thirds or 66.5% of the national income (CEPAL, 2018, p. 62), making the lower classes especially susceptible to fall into poverty (Denis et al., 2007; Maldonado & Prieto, 2015). The minimum wage in Chile between 2016 and 2021 increased from $250,000 Chilean peso (CLP) in January 2016 to $337,000 in May of 2021 (BCN, 2014, 2021), with $100,000 CLP estimated to be approximately $140 US dollars when averaging the exchange rate from 2016 to 2021 (Banco Central, 2022).

This context of constant, though unequal economic growth, and a rapid increase in immigration from relatively similar countries, offers a good opportunity for studying to what extent education matters for anti‐immigrant sentiment associated with perceived economic and cultural threat. It provides a new setting that helps to delineate possible scope conditions of the universality of the so‐called “liberalizing” effect of education and generalizability of results beyond the global North, when considering most studies have focused on Western countries, which have considerably lower levels of inequality and more diverse immigrant ethnic compositions.

In order to establish the link of whether education is a determinant of anti‐immigrant attitudes, and to what extent this relationship is associated with immigrants as a perceived economic or cultural threat, I first hypothesize that:

H 1a Individuals who have higher levels of education are less likely to perceive immigrants as an economic threat than individuals with lower levels of education.

H 1b Individuals who have higher levels of education are less likely to perceive immigrants as a cultural threat than individuals with lower levels of education.

I then take advantage of the longitudinal nature of the data and put forward hypotheses to explore *if* attaining more education decreases prejudice, speaking to the causal effect of education on attitudes:

H 2a Attaining more education leads to less perceived economic threat.

H 2b Attaining more education leads to less perceived cultural threat.

## 4 DATA

ELSOC has been administered annually in the years 2016–2022 with the exception of 2020 due to the COVID‐19 pandemic. It is the first panel study in Latin America, with a representative sample of the Chilean urban population aged 18 and older and reaches a representativeness of 77% of the country as a whole, and 93% of the urban population (ELSOC, 2023). In the original sample, 2927 respondents were surveyed in the first wave, with an attrition rate of 15.5% for the second wave; 9.9% for the third wave; 3.4% for the fourth wave; 19.2% for the fifth wave; and 0.6% for the last wave. In order to counteract the attrition rate, refreshment samples were added starting in the third wave (2018). In this study, the number of respondents for waves 1–6 is: 2750; 2384; 3537; 3334; 2689; 2666. In total, the size of analytical sample for this study is 4275 individuals and 17,360 observations.

## 5 DEPENDENT VARIABLES

Two dependent variables are asked in all waves, which I label “perceived economic threat” and “perceived cultural threat.” Both variables are Likert scales that capture the level of disagreement‐agreement with two statements. Perceived economic threat is captured by the statement: “With the arrival of many [Peruvians/Haitians/Venezuelans] in Chile, unemployment is increasing.” The second dependent variable is labeled “perceived cultural threat,” and similarly captures level of disagreement‐agreement with the statement: “With the arrival of many [Peruvians/Haitians/Venezuelans], Chile is losing its identity.” For both variables, higher values reflect more negative attitudes toward immigrants (more perceived threat): 0 “Completely disagree,” 1 “Disagree” 2 “Neither disagree nor agree,” 3 “Agree,” and 4 “Completely agree.” In waves 1 and 2, respondents were only asked about Peruvian immigrants. In wave 3 (refreshment sample), respondents were asked either about Peruvian or Haitian immigrants, and in the following waves, respondents were asked about their attitudes toward either Peruvian, Haitian, or Venezuelan immigrants. In the original data from ELSOC (2023), these answers are treated similarly and are used to infer general attitudes toward the three largest immigrant groups in the country. Figures A1–A7 in Appendix show the time‐trends for the responses for perceived economic and cultural threat for each ethnic group asked in the survey, as well as by respondents' educational level. The figures show similar trends, although answers regarding Venezuelans in both questions seem to diverge slightly from the other two groups, especially in the last wave.

## 6 INDEPENDENT VARIABLES

### 6.1 Main independent variable

The main independent variable is highest level of education. In the models, it is used both as a categorical variable to establish the inverse relationship between education and prejudice and as a continuous variable for purposes of mean‐centering (see Section 7). In the original data, the education variable consists of 10 categories, where I merge the first two categories into one (“Primary incomplete/No studies”) resulting in a 9‐category educational variable: “Primary incomplete/No studies”; “Primary education”; “Secondary incomplete”; “Secondary education”; “Technical incomplete”; “Technical education”; “University incomplete”; “University education”; “Postgraduate studies.” Respondents with “incomplete” education are those currently studying or have begun their studies but have not completed them, yet. Additionally, in order to investigate transitions in education and whether this leads to less perceived threat, I merge these categories into a 4‐category variable: “Primary education or less”; “Secondary education”; “Technical education”; “University education.” Respondents with “incomplete” education are placed in the educational level they began (e.g., secondary incomplete are under secondary education). Approximately 29% of respondents in this sample experienced any type of increase in education (e.g., incomplete‐complete, incomplete‐incomplete, complete‐incomplete transitions, etc.) accounting for almost 32% of the observations. Educational transitions represent 16% of respondents in the sample (using the 4‐category variable), and 18% of the observations. A breakdown of actual changes in educational attainment in the sample according to age‐group, type of transition and wave are found in Tables A1–A5 in Appendix.

### 6.2 Controls

As basic demographic controls, I use a binary variable for sex, and a continuous variable for age. In order to isolate the effect of education on attitudes, I include two socioeconomic controls: total monthly household income and employment status. Income is included as a 9‐category variable with increments of $250,000 CLP or one minimum‐wage salary in 2016. This variable is censored at $2,000,000 or more, or eight times the minimum‐wage salary in 2016. A continuous variable for income is included for mean‐centering purposes and is censored at $9,000,000 CLP with observations of respondents earning more having been included in this value (<30). Graphs of these variables are included in Figures A8 and A9 in Appendix. Employment status is recorded from a 9‐category variable to a 7‐category one: “Working full‐time”; “Working part‐time”; “Work and studies”; “Only studies”; “Retired”; “Unemployed”; and “Stay‐at‐home.” The category unemployed includes respondents who are “unemployed and looking for jobs”; “not working, studying, nor looking for jobs”; and “disabled or sick.”

## 7 METHODS

I analyze data with mixed, multilevel repeated measurement models. These are hierarchical models which are appropriate when clustering in the data is present, such as in this case, with multiple observations by the same individuals over time. Failing to recognize these hierarchical structures can lead to the underestimation of standard errors, since observations are non‐independent, which can lead to an overestimation of statistical significance and type I errors. This approach estimates fixed‐effects and two random‐effects, corresponding to between‐individual (intercepts) and within‐individual (residuals) variances. The generic model using a single equation looks like this:

$$y_{ij\;}=\beta_{0}+\beta_{1}x_{1\;}+\beta_{2}\;\left(x_{ij\;}-{\bar{x}}_{j}\right)+{\;\beta }_{3}{\bar{x}}_{j}\;\text{…}\;\beta_{n}x_{n}+u_{j\;}+e_{ij\;}$$

where *y*_*ij*_ is the dependent variable measured for *i* th level‐1 unit (observations) nested within the *j* th level‐2 unit (individuals). The fixed‐part of the model consists of the intercept *β*_0_ and covariates *β*_1_ *x*_1_ +… *β*_*n*_ *x*_*n*_. The random‐part consists of *u*_*j*_, the residual variance of the level‐2 units (individuals) and *e*_*ij*_, the residual variance of the level‐1 units (observations). In order to separate within‐from between‐individual educational effects (as well as income), I use mean‐centering (Allison, 2009; Fairbrother, 2014) to create two variables for each respondent: the mean of an individual's response over time (*b*) and the difference from the mean for the response at each time point (*w*). This is captured by the *β*_2_(*x*_*ij*_ − ${\bar{x}}_{j}$) + ${\;\beta }_{3}{\bar{x}}_{j}$ part of the model, with the first coefficient representing the within‐individual effects (difference from an individual's average response over time) and the latter represents the between‐individual effects (mean of an individual's responses over time). This approach is called a random‐effect‐within‐between model or “REWB,” with heterogeneity modeled at both the cluster (level 2) and observation (level 1) level, allowing for distinct between‐ and within‐individual effects (Bell et al., 2019). By including both variables in the same model, I can assess whether higher levels of education are associated with lower levels of anti‐immigrant sentiment, as well as whether *attaining* more education has an impact on these attitudes over time.

## 8 RESULTS

### 8.1 Descriptive statistics

Table 1 reports descriptive findings of the data. The most common type of completed education among respondents in the sample is secondary education with 29%. This is followed by technical education (15%), university education (13%), primary education (9%), and postgraduate studies (3%). The rest report their current level of education as “incomplete.” The sample is skewed with more females (62%) than males (38%), with the average age of respondents being 48. The average monthly household income is $713,000 CLP, or slightly lower than three minimum‐wage salaries in 2016. Almost half of the sample indicated working full‐time (44%), working part‐time (16%), retired (15%) stay‐at‐home (12%), unemployed (9%), only studying (3%), and working and studying (2%).

**TABLE 1:** Descriptive statistics.

	Mean/%	SD	Min	Max
Dependent variables				
Perceived economic threat	2.22	1.17	0	4
Perceived cultural threat	1.92	1.16	0	4
Main independent variable				
Highest level of education				
Primary incomplete/no studies	0.08	0.27	0	1
Primary	0.09	0.29	0	1
Secondary incomplete	0.12	0.33	0	1
Secondary	0.29	0.45	0	1
Technical incomplete	0.04	0.19	0	1
Technical	0.15	0.36	0	1
University incomplete	0.07	0.26	0	1
University	0.13	0.34	0	1
Postgraduate studies	0.03	0.16	0	1
Demographic controls				
Male	0.38	0.49	0	1
Female	0.62	0.49	0	1
Age	48.3	15.4	18	92
Socioeconomic controls				
Household income (100,000 CLP)	7.13	8.15	0	90
Employment status				
Work full‐time	0.44	0.50	0	1
Work part‐time	0.16	0.36	0	1
Work and studies	0.02	0.15	0	1
Only studies	0.03	0.16	0	1
Retired	0.15	0.35	0	1
Unemployed	0.09	0.28	0	1
Stay‐at‐home	0.12	0.33	0	1

Abbreviation: CLP, Chilean peso.

*Source*: Chilean Longitudinal Social Survey (ELSOC) 2016–2022. *N* = 4275.

### 8.2 Multilevel analyses

In Table 2, I report results from multilevel repeated measurement analyses for the “perceived economic threat” dependent variable in models 1–6. I begin with model 1, including a linear time variable to rule out a possible correlation due to common trending, and the 9‐category education variable, in order to examine whether education follows a linear pattern. This model shows that higher levels of education are associated with less perceived economic threat as compared to respondents with a primary education or less. That is, respondents with higher levels of education are less likely to respond that unemployment is increasing with the arrival of many immigrants. In model 2, I include employment status, age, and sex as controls. This model shows that respondents who work part‐time and are unemployed are more likely to respond that unemployment is increasing with the arrival of immigrants, as compared to those working full‐time. Respondents who are working and studying, or only studying are less likely to respond immigrants are a cause for rising unemployment. In model 3, I include the 9‐category income variable, showing that respondents whose household income is $750,000 CLP or above are more likely to disagree with the statement that immigrants increase unemployment as compared to those with the lowest household incomes, with the exception of those who earn between $1,500,000–1,749,999 CLP. In models 4–6, the mean for individuals over time (b) and the difference from the mean (*w*) are shown for education and income, with and without controls. These models show a weak but statistically significant within‐individual effect of attaining more education on perceived economic threat. Put differently, those who attain more education during this period are less likely to respond that unemployment increases because of immigration. Attaining more income, however, does not have an effect on perceived economic threat.

**TABLE 2:** Multilevel repeated measurement models (perceived economic threat).

	Perceived economic threat					
	(1)	(2)	(3)	(4)	(5)	(6)
Time	−0.006	−0.014**	−0.011*	−0.017***	−0.022***	−0.020***
	(0.005)	(0.005)	(0.005)	(0.005)	(0.005)	(0.005)
Education (ref. primary incomplete/no studies)						
Primary	−0.108*	−0.092	−0.091			
	(0.049)	(0.049)	(0.049)			
Secondary incomplete	−0.136**	−0.095*	−0.089			
	(0.048)	(0.048)	(0.048)			
Secondary	−0.321***	−0.255***	−0.238***			
	(0.043)	(0.044)	(0.045)			
Technical incomplete	−0.535***	−0.401***	−0.372***			
	(0.064)	(0.067)	(0.067)			
Technical	−0.524***	−0.447***	−0.409***			
	(0.048)	(0.049)	(0.050)			
University incomplete	−0.743***	−0.560***	−0.518***			
	(0.054)	(0.059)	(0.060)			
University	−0.948***	−0.853***	−0.769***			
	(0.049)	(0.051)	(0.054)			
Postgraduate studies	−1.235***	−1.145***	−1.026***			
	(0.078)	(0.079)	(0.082)			
Education‐level (b)				−0.150***	−0.137***	−0.116***
				(0.006)	(0.006)	(0.007)
Education‐level (w)				−0.042*	−0.041*	−0.041*
				(0.017)	(0.017)	(0.017)
Income (b)						−0.013***
						(0.002)
Income (w)						−0.003
						(0.002)
Income (ref. 249,999 CLP or less)						
250,000–499,999			0.006			
			(0.028)			
500,000–749,999			−0.042			
			(0.031)			
750,000–999,999			−0.097*			
			(0.039)			
1,000,000–1,249,999			−0.129**			
			(0.042)			
1,250,000–1,499,999			−0.185**			
			(0.067)			
1,500,000–1,749,999			−0.047			
			(0.055)			
1,750,000–1,999,999			−0.260**			
			(0.086)			
2,000,000 or more			−0.229***			
			(0.052)			
Main activity (ref. work full‐time)						
Work part‐time		0.068**	0.053*		0.065*	0.054*
		(0.026)	(0.026)		(0.026)	(0.026)
Work and studies		−0.119*	−0.136*		−0.090	−0.105
		(0.059)	(0.059)		(0.057)	(0.057)
Only studies		−0.201**	−0.227***		−0.137*	−0.162**
		(0.062)	(0.062)		(0.059)	(0.059)
Retired		0.055	0.026		0.054	0.028
		(0.035)	(0.036)		(0.035)	(0.035)
Unemployed		0.094**	0.067*		0.094**	0.076*
		(0.032)	(0.032)		(0.032)	(0.032)
Stay‐at‐home		0.053	0.035		0.053	0.041
		(0.031)	(0.032)		(0.031)	(0.031)
Female		0.048	0.036		0.046	0.032
		(0.025)	(0.026)		(0.026)	(0.025)
Age		0.004***	0.004***		0.003**	0.003**
		(0.001)	(0.001)		(0.001)	(0.001)
Constant	2.660***	2.377***	2.415***	2.807***	2.592***	2.614***
	(0.039)	(0.066)	(0.069)	(0.026)	(0.058)	(0.058)
Random‐effects parameters						
Between‐individual	0.368***	0.362***	0.358***	0.372***	0.366***	0.360***
	(0.013)	(0.013)	(0.013)	(0.013)	(0.013)	(0.013)
Within‐individual	0.898***	0.896***	0.895***	0.896***	0.895***	0.895***
	(0.011)	(0.011)	(0.011)	(0.011)	(0.011)	(0.011)
log Likelihood	−25718.043	−25684.994	−25665.074	−25716.627	−25693.303	−25670.605
Observations	17,360	17,360	17,360	17,360	17,360	17,360
Individuals	4275	4275	4275	4275	4275	4275

*Note*: Standard errors in parentheses.

Abbreviation: CLP, Chilean peso.

***p < 0.001, **p < 0.01, *p < 0.05.

*Source*: Chilean Longitudinal Social Survey (ELSOC) 2016–2022.

Similar analyses were conducted for the “perceived cultural threat” dependent variable in Table 3. In model 7, I add the continuous time variable together with the 9‐category education variable. This model reports that every increase in education contributes to lower levels of perceived cultural threat, comparable to the previous analyses, although the effects are slightly larger. In model 8, I control for employment status, age, and sex, showing that those who are currently studying are less likely to respond that Chile is losing its identity due to the arrival of many immigrants. In model 9, I add the 9‐category income variable and it shows that those earning $1,000,000 CLP or more (except household incomes of 1,500,000–1,749,999 CLP) are likely to report lower levels of cultural threat compared to those with the lowest household incomes. However, this association is weaker than in the analyses in model 3. Models 10–12 report between‐ and within‐individual effects of education and income, without and with controls. Similarly, as in models 4–6, these three models provide evidence that attaining more education makes individuals less likely to respond that Chile is losing its identity due to the arrival of immigrants, and this effect holds even when controlling for other individual‐level factors. Increases in income, however, do not have an effect on perceived cultural threat.

**TABLE 3:** Multilevel repeated measurement models (perceived cultural threat).

	Perceived cultural threat					
	(7)	(8)	(9)	(10)	(11)	(12)
Time	0.027***	0.022***	0.024***	0.018***	0.014**	0.015**
	(0.005)	(0.005)	(0.005)	(0.005)	(0.005)	(0.005)
Education (ref. primary incomplete/no studies)						
Primary	−0.162***	−0.149**	−0.152**			
	(0.048)	(0.048)	(0.048)			
Secondary incomplete	−0.192***	−0.161***	−0.163***			
	(0.047)	(0.047)	(0.048)			
Secondary	−0.380***	−0.331***	−0.324***			
	(0.042)	(0.043)	(0.044)			
Technical incomplete	−0.574***	−0.479***	−0.460***			
	(0.063)	(0.066)	(0.066)			
Technical	−0.585***	−0.530***	−0.501***			
	(0.047)	(0.048)	(0.049)			
University incomplete	−0.818***	−0.687***	−0.652***			
	(0.053)	(0.058)	(0.059)			
University	−0.920***	−0.851***	−0.773***			
	(0.048)	(0.050)	(0.053)			
Postgraduate studies	−1.154***	−1.086***	−0.969***			
	(0.076)	(0.077)	(0.080)			
Education‐level (b)				−0.141***	−0.131***	−0.112***
				(0.005)	(0.006)	(0.007)
Education‐level (w)				−0.055**	−0.054**	−0.054**
				(0.017)	(0.017)	(0.017)
Income (b)						−0.012***
						(0.002)
Income (w)						−0.001
						(0.002)
Income (ref. 249,999 CLP or less)						
250,000–499,999			0.049			
			(0.028)			
500,000–749,999			0.009			
			(0.032)			
750,000–999,999			−0.046			
			(0.039)			
1,000,000–1,249,999			−0.104*			
			(0.043)			
1,250,000–1,499,999			−0.236***			
			(0.068)			
1,500,000–1,749,999			0.009			
			(0.055)			
1,750,000–1,999,999			−0.182*			
			(0.086)			
2,000,000 or more			−0.199***			
			(0.052)			
Main activity (ref. work full‐time)						
Work part‐time		0.004	−0.008		0.001	−0.009
		(0.026)	(0.026)		(0.026)	(0.026)
Work and studies		−0.094	−0.111		−0.091	−0.104
		(0.059)	(0.059)		(0.057)	(0.057)
Only studies		−0.153*	−0.174**		−0.132*	−0.154**
		(0.063)	(0.063)		(0.059)	(0.059)
Retired		0.051	0.031		0.050	0.025
		(0.035)	(0.036)		(0.035)	(0.035)
Unemployed		0.037	0.016		0.037	0.021
		(0.032)	(0.033)		(0.032)	(0.032)
Stay‐at‐home		0.054	0.040		0.052	0.042
		(0.031)	(0.032)		(0.031)	(0.031)
Female		0.067**	0.057*		0.067**	0.054*
		(0.025)	(0.025)		(0.025)	(0.025)
Age		0.002*	0.002*		0.002	0.002*
		(0.001)	(0.001)		(0.001)	(0.001)
Constant	2.322***	2.125***	2.126***	2.392***	2.231***	2.252***
	(0.038)	(0.064)	(0.068)	(0.026)	(0.056)	(0.056)
Random‐effects parameters						
Between‐individual	0.317***	0.312***	0.308***	0.318***	0.313***	0.308***
	(0.013)	(0.012)	(0.012)	(0.013)	(0.012)	(0.012)
Within‐individual	0.938***	0.937***	0.936***	0.936***	0.937***	0.936***
	(0.012)	(0.012)	(0.012)	(0.012)	(0.012)	(0.012)
log Likelihood	−25858.810	−25839.553	−25814.091	−25855.190	−25838.133	−25817.708
Observations	17,360	17,360	17,360	17,360	17,360	17,360
Individuals	4275	4275	4275	4275	4275	4275

*Note*: Standard errors in parentheses.

Abbreviation: CLP, Chilean peso.

***p < 0.001, **p < 0.01, *p < 0.05.

*Source*: Chilean Longitudinal Social Survey (ELSOC) 2016–2022.

Tables 4 and 5 show general categories for educational transitions using fixed‐effects models, which take into account within‐individual differences while ignoring between‐individual differences. Each model is estimated separately for each origin category while controlling for age and income.

**TABLE 4:** Perceived economic threat: Educational transitions.

	*b*	SE	*N*	*N*	*p*‐value	Model
			Individuals	Observations		
Primary → secondary	−0.035	(0.087)	207	937	.690	13
Secondary → technical	−0.131	(0.079)	255	1141	.097	14
Secondary → university	−0.188	(0.135)	87	343	.164	15
Technical → university	−0.160	(0.100)	146	641	.109	16

*Note*: Models estimated separately for each origin category (controls: age and income).

***p < 0.001, **p < 0.01, *p < 0.05.

*Source*: Chilean Longitudinal Social Survey (ELSOC) 2016–2022.

**TABLE 5:** Perceived cultural threat: Educational transitions.

	*b*	SE	*N*	*N*	*p*‐value	Model
			Individuals	Observations		
Primary → secondary	0.127	(0.091)	207	937	.163	17
Secondary → technical	−0.124	(0.081)	255	1141	.128	18
Secondary → university	−0.249	(0.127)	87	343	.052	19
Technical → university	−0.064	(0.096)	146	641	.507	20

*Note*: Models estimated separately for each origin category (controls: age and income).

***p < 0.001, **p < 0.01, *p < 0.05.

*Source*: Chilean Longitudinal Social Survey (ELSOC) 2016–2022.

Models 13–16 report the effect of educational transitions on perceived economic threat. Although these are not statistically significant, the lowest *p*‐value corresponds to the transition from secondary to technical education (*p* = 0.097). In models 17–20, similar analyses were conducted for perceived cultural threat. Model 19, although not statistically significant, shows that attaining a university education is likely to be driving the relationship in the main analyses (*p* = 0.052).

## 9 CONCLUSION

Although a plethora of studies have repeatedly shown an inverse relationship between education and prejudice, this well‐established finding has not been thoroughly investigated in a non‐Western context. Further, very few studies have examined whether this relationship is causal, mainly due to a lack of longitudinal studies. In this paper, I have analyzed the effects of education on attitudes toward immigrants (perceived economic threat and perceived cultural threat) using longitudinal data from Chile, a country in Latin America that in recent years has experienced a rapid growth in its economy, accompanied by increased immigration.

I devised two sets of hypotheses. First, to establish the empirical link between higher levels of education and lower levels of prejudice, I hypothesized that individuals who have higher levels of education are less likely to perceive immigrants as an economic threat (H1a) and cultural threat (H2a) than individuals with lower levels of education. The results show support for both hypotheses. In other words, individuals with higher levels of education are less likely to respond that either unemployment is increasing, or that Chile is losing its identity with the arrival of many immigrants. Second, taking advantage of the longitudinal nature of the data and speaking to the causal effect of education on attitudes, I hypothesized that attaining more education leads to less perceived economic threat (H1b) and less perceived cultural threat (H2b). Attaining more education during this period is shown to be associated with lower levels of both perceived economic threat and perceived cultural threat, providing support for hypothesis H2a and H2b, with education having a stronger effect on the latter.

Even though I cannot distinguish among the possible theoretical mechanisms responsible for this relationship, the results indicate a potential liberalizing effect of education to have a stronger effect on attitudes toward immigrants when these concern immigrants' cultural impact on the country as a whole, and a weaker effect in regard to their economic impact. This is further confirmed with additional analyses similar to those in Tables 2 and 3 but using the 4‐category education variable (Tables A8 and A9 in Appendix), showing within‐individual effects of education on perceived economic threat disappear. A possible explanation behind these divergencies can be the age composition of the sample. The majority of respondents experiencing educational transitions are over 30 years old (Table A1 in Appendix), which could potentially be underestimating the liberalizing effect of education on these attitudes, since older people are less susceptible to attitudinal change (Krosnick & Alwin, 1989). Lastly, results from the fixed‐effects models, suggest that educational effects are likely to be driven by the educational transition from secondary to university education in the case of perceived cultural threat, and from secondary to technical education in the case of perceived economic threat. This hints to socialization and academic field as potentially responsible for this difference (e.g., Eger et al., 2024). While these results are not conclusive, they speak to general trends in Western nations, where attitudes toward immigration are increasingly shaped by its cultural impacts (Hainmueller & Hopkins, 2014).

Still, economic concerns in Chile are still important in relation to individuals' personal economic circumstances and the impact of immigrants on the economy. For example, respondents who work part‐time and are unemployed are more likely to believe unemployment is increasing due to the arrival of immigrants, compared to those who work full‐time. Further, individuals who have incomplete secondary education or less, are as likely to believe unemployment is increasing due to the arrival of immigrants, as compared to those with the lowest level of education, whereas every increase in education is associated with lower perceived cultural threat. In other words, respondents who have very low levels of education are likely to perceive immigrants as an economic threat, and may believe themselves to be in direct competition with them or blame immigrants for their situation in the labor market (e.g., Ajzenman et al., 2022). This may be particularly true when considering immigrants in Chile are, on average, more educated than the Chilean population, having two more years of schooling than the Chilean population aged 18 and older (Baeza Virgilio, 2019, p. 5).

Lastly, (household) income is still a strong determinant in explaining perceived economic and cultural threat. This may be attributed to parental status, income, and education being very strong structural determinants of access to higher education in Chile (Bazoret, 2006; Espinoza & González, 2013; González et al., 2016), where higher education is the second most expensive of any OECD country, with approximately 70% attending private institutions (Arango et al., 2016; Brunner & Labraña, 2020). That is, individuals who attend higher education are more likely to come from households with higher income and education. Attaining more education could then be interpreted to be an economic gain in regard to one's position in the social structure as but also as a cultural gain in one's values related to diversity, as evidenced by the stronger relationship between education and less perceived cultural threat.

A few limitations of this study should be acknowledged. First, starting in the third wave, respondents start being asked about additional immigrant groups. This could have some implications in terms of attitudinal trends toward different groups over time. Although visual representations of these trends (Figure A1 in Appendix) show that there are slight differences on how these immigrant groups are viewed, they show no substantial divergences by level of education (Figures A2–A7). This lends support to the idea that respondents view immigrant groups similarly or at the very least, not radically different (Goubin & Ruelens, 2024). A second point is that while studies on prejudice often rely on composite scales measuring different aspects of perceived ethnic threat (e.g., Hjerm, 2001; Manevska & Achtenberg, 2013; Schneider, 2008), this study uses two dependent variables. Using single‐measure items may be less stable than multiple‐item scales, although this is a point of contention (e.g., Bergkvist & Rossiter, 2007; Diamantopoulos et al., 2012; Matthews et al., 2022). Despite this, further analyses were conducted combining both dependent variables into an index, showing similar results as the ones provided in the main analyses (Tables A6 and A7 in Appendix).

In conclusion, this study provides a new setting of high inequality and objectively similar immigrants in an effort to delineate possible scope conditions of the so‐called “liberalizing” effect of education and generalizability of results beyond the global North. To answer the first question posed earlier: does attaining more education reduce anti‐immigrant sentiment? I show that this is the case, thus lending support to the potential liberalizing effect of education. Higher *levels* of education and *attaining* more education are associated with both less perceived economic threat and less perceived cultural threat, with education having a stronger effect on the latter. To answer the second question: why might this be different in a country in the global South? It may not be so different, after all. I provide empirical evidence from an interrupted democracy in a non‐Western context, displaying similar results from studies conducted in North America and Europe by showing an inverse relationship between education and prejudice. Indeed, literature on the democratic learning hypothesis (Marquart‐Pyatt & Paxton, 2007; Weil, 1989) has shown that the longer the democratic tradition of a country, the stronger the effect of education is on liberal values. In contrast, the impact of education on liberal values is weaker, insignificant or even reversed in non‐liberal democracies or countries with a short democratic tradition (Hello et al., 2002; Weil, 1985). This study yields support for this hypothesis, yet it remains to be shown whether this educational effect is weaker or stronger in Chile than in other countries with more prolonged democratic traditions or more recent ones (e.g., Coenders & Scheepers, 2003).

Lastly, even though this study shows that educational attainment alleviates concerns for immigrants' economic and cultural impact in the country, it also shows that personal economic circumstances do correlate with perceived economic threat, which has repeatedly failed to receive empirical support in Western nations (Hainmueller & Hopkins, 2014, p. 241). Nonetheless, while education is correlated with income and class, it seems to be increasingly exhibiting distinct properties as evidenced by a stronger effect on perceived cultural threat. This may imply that in Chile, as in European societies, education is becoming a better predictor for different attitudes than economic/class indicators (Kalmijn & Kraaykamp, 2007). All in all, this suggests that (attaining) education should likely be treated as a cultural variable (Manevska & Achterberg, 2013), while taking into consideration that economic concerns are still meaningful in Chile.

## CONFLICT OF INTEREST STATEMENT

The authors declare no conflicts of interest.

## Supporting information

Supplementary Material

## Data Availability

The data that support the findings of this study are openly available in Reproducible Research, Centre for Social Conflict and Cohesion Studies COES, 2023, “Estudio Longitudinal Social de Chile 2016–2022,” https://doi.org/10.7910/DVN/QZEDUC, Harvard Dataverse, V3.

## ENDNOTES

^1^
Full list included: Mapuche (indigenous), disabled, evangelicals, and the elderly.

^2^
Chile has an estimated number of immigrants in the country in 2022, but no national census has been conducted since 2017. This means that this percentage is likely to be an overestimation, although not a large one.

## References

[bjos13124-bib-0001] Ajzenman, N. , Dominguez, P. , & Undurraga, R. (2022). Immigration and labor market (mis)perceptions. AEA Papers and Proceedings, 112, 402–408. 10.1257/pandp.20221004

[bjos13124-bib-0002] Allison, P. D. (2009). Fixed effects regression models. SAGE Publications.

[bjos13124-bib-0003] Arango, M. , Evans, S. , & Quadri, Z. (2016). Education reform in Chile: Designing a fairer, better higher education system. Graduate Policy Workshop Woodrow Wilson School of Public & International Affairs, Princeton University.

[bjos13124-bib-0004] Baeza Virgilio, P. (2019). Incorporación de inmigrantes sudamericanos en Santiago de Chile: redes migratorias y movilidad ocupacional [Incorporation of South American immigrants in Santiago de Chile: Migratory networks and occupational mobility]. Migraciones Internacionales, 10(8), 1–27. 10.33679/rmi.v1i1.2145

[bjos13124-bib-0005] Banco Central [Central Bank] . (2022). Tipos de cambio [Exchange rates]. Retrieved July 28, 2022, from https://si3.bcentral.cl/Siete/ES/Siete/Cuadro/CAP_TIPO_CAMBIO/MN_TIPO_CAMBIO4/DOLAR_OBS_ADO

[bjos13124-bib-0006] Bazoret, E. (2006). El Valor Historico del Pituto: Clase Media, Integracion y Diferenciación Social en Chile. Revista de sociología, 20, 69–96.

[bjos13124-bib-0007] BCN‐Biblioteca del Congreso Nacional de Chile [Library of the National Congress of Chile] . (2014). Retrieved July 28, 2022, from https://www.bcn.cl/leychile/navegar?idLey=20763

[bjos13124-bib-0008] BCN‐Biblioteca del Congreso Nacional de Chile [Library of the National Congress of Chile] . (2021). Retrieved July 28, 2022, from https://www.bcn.cl/leychile/navegar?idNorma=1162462

[bjos13124-bib-0009] Bell, A. , Fairbrother, M. , & Jones, K. (2019). Fixed and random effects models: Making an informed choice. Quality and Quantity, 53(2), 1051–1074. 10.1007/s11135-018-0802-x

[bjos13124-bib-0010] Bergkvist, L. , & Rossiter, J. R. (2007). The predictive validity of multiple‐item versus single‐item measures of the same constructs. Journal of Marketing Research, 44(2), 175–184. 10.1509/jmkr.44.2.175

[bjos13124-bib-0011] Blumer, H. (1958). Race prejudice as a sense of group position. Pacific Sociological Review, 1(1), 3–7. 10.2307/1388607

[bjos13124-bib-0012] Bobo, L. , & Licari, F. C. (1989). Education and political tolerance: Testing the effects of cognitive sophistication and target group affect. Public Opinion Quarterly, 53(3), 285–308. 10.1086/269154

[bjos13124-bib-0013] Bonhomme, M. (2023). ‘We’re a bit browner but we still belong to the white race’: Making whiteness in the context of South‐South migration in Chile. Latin American and Caribbean Ethnic Studies, 18(2), 227–243. 10.1080/17442222.2022.2099170

[bjos13124-bib-0014] Braun, J. , Braun, M. , Briones, I. , Díaz, J. , Lüders, R. , & Wagner, G. (2000). Economía chilena 1819–1995: Estadísticas históricas. Pontificia Universidad Católica de Chile.

[bjos13124-bib-0015] Brunner, J. J. , & Labraña, J. (2020). The transformation of higher education in Latin America: From elite access to massification and universalisation. In S. Schwartz (Ed.), Higher education in Latin America and the challenges of the 21st century (pp. 31–41). Springer.

[bjos13124-bib-0016] Carvacho, H. , Zick, A. , Haye, A. , González, R. , Manzi, J. , Kocik, C. , & Bertl, M. (2013). On the relation between social class and prejudice: The roles of education, income, and ideological attitudes. European Journal of Social Psychology, 43(4), 272–285. 10.1002/ejsp.1961

[bjos13124-bib-0017] Castro Torres, A. F. , & Alburez‐Gutierrez, D. (2022). North and South: Naming practices and the hidden dimension of global disparities in knowledge production. Proceedings of the National Academy of Sciences, 119(10), e2119373119. 10.1073/pnas.2119373119 PMC8915996 35238625

[bjos13124-bib-0018] Ceobanu, A. M. , & Escandell, X. (2010). Comparative analyses of public attitudes toward immigrants and immigration using multinational survey data: A review of theories and research. Annual Review of Sociology, 36(1), 309–328. 10.1146/annurev.soc.012809.102651

[bjos13124-bib-0019] CEPAL‐Comisión Económica para América Latina y el Caribe [Economic Commission for Latin America and the Caribbean] . (2018). Panorama Social de América Latina. CEPAL. Retrieved August 3, 2022, from https://www.cepal.org/es/publicaciones/44395‐panorama‐social‐america‐latina‐2018

[bjos13124-bib-0020] Citrin, J. , Green, D. P. , Muste, C. , & Wong, C. (1997). Public opinion toward immigration reform: The role of economic motivations. The Journal of Politics, 59(3), 858–881. 10.2307/2998640

[bjos13124-bib-0021] Coenders, M. , & Scheepers, P. (2003). The effect of education on nationalism and ethnic exclusionism: An international comparison. Political Psychology, 24(2), 313–343. 10.1111/0162-895x.00330

[bjos13124-bib-0022] Denis, A. , Prieto, J. J. , & Zubizarreta, J. R. (2007). Dinámica de la pobreza en Chile: evidencias en los años 1996, 2001 y 2006 [The dynamics of poverty in Chile: Findings in 1996, 2001 and 2006]. Persona y Sociedad, 21(3), 9. 10.53689/pys.v21i3.149

[bjos13124-bib-0023] Diamantopoulos, A. , Sarstedt, M. , Fuchs, C. , Wilczynski, P. , & Kaiser, S. (2012). Guidelines for choosing between multi‐item and single‐item scales for construct measurement: A predictive validity perspective. Journal of the Academy of Marketing Science, 40(3), 434–449. 10.1007/s11747-011-0300-3

[bjos13124-bib-0024] Dražanová, L. (2017). Education and tolerance: A comparative quantitative analysis of the educational effect on tolerance. Peter Lang.

[bjos13124-bib-0025] Dražanová, L. , Gonnot, J. , Heidland, T. , & Krüger, F. (2023). Which individual‐level factors explain public attitudes toward immigration? A meta‐analysis. Journal of Ethnic and Migration Studies, 50(2), 1–24. 10.1080/1369183x.2023.2265576

[bjos13124-bib-0026] Eger, M. A. , Hjerm, M. , & Velásquez, P. (2024). Unpacking the liberalizing potential of higher education: An analysis of academic majors, anti‐Black prejudice, and opposition to immigration. Ethnic and Racial Studies, 1–36. 10.1080/01419870.2023.2295479

[bjos13124-bib-0027] ELSOC‐Estudio Longitudinal Social de Chile 2016‐2022[Chilean Longitudinal Social Survey] . (2023). Reproducible research, Centre for social conflict and Cohesion studies (COES). Harvard Dataverse, V3. 10.7910/DVN/QZEDUC

[bjos13124-bib-0028] Espinoza, O. , & González, L. E. (2013). Access to higher education in Chile: A public vs. private analysis. Prospects, 43(2), 199–214. 10.1007/s11125-013-9268-8

[bjos13124-bib-0029] Fairbrother, M. (2014). Two multilevel modeling techniques for analyzing comparative longitudinal survey datasets. Political Science Research and Methods, 2(01), 119–140. 10.1017/psrm.2013.24

[bjos13124-bib-0030] Fetzer, J. S. (2000). Economic self‐interest or cultural marginality? Anti‐Immigration sentiment and nativist political movements in France, Germany and the USA. Journal of Ethnic and Migration Studies, 26(1), 5–23. 10.1080/136918300115615

[bjos13124-bib-0031] Fuentes, A. , & Hernando, A. (2020). CARACTERIZACIÓN ESTADÍSTICA DE LA INMIGRACIÓN EN CHILE [Statistics on immigration to Chile]. In I. Aninat & R. Vergara (Eds.), Inmigración en Chile. Una mirada multidimensional [Immigration in Chile. A multidimensional view] (2nd ed., pp. 411–439). FCE, CEP.

[bjos13124-bib-0032] González, R. (2005). Movilidad social: El rol del prejuicio y la discriminación. En Foco, 59, 1–23.

[bjos13124-bib-0033] González, R. , Gerber, M. M. , & Carvacho, H. (2016). Social identities and conflict in Chile: The role of historical and political processes. In S. McKeown , R. Haji , & N. Ferguson (Eds.), Understanding peace and conflict through social identity theory (1st ed., pp. 3–17). Springer International Publishing.

[bjos13124-bib-0034] Goubin, S. , & Ruelens, A. (2024). Towards a typology of European migration attitudes across time and space: A person‐centred approach. Journal of Ethnic and Migration Studies, 1–22. 10.1080/1369183x.2023.2301406

[bjos13124-bib-0035] Hagendoorn, L. , & Nekuee, S. (1999). Education and racism: A cross national inventory of positive effects of education on ethnic tolerance. Ashgate Publishing.

[bjos13124-bib-0036] Hainmueller, J. , & Hiscox, M. J. (2007). Educated preferences: Explaining attitudes toward immigration in Europe. International Organization, 61(2), 399–442. 10.1017/s0020818307070142

[bjos13124-bib-0037] Hainmueller, J. , & Hopkins, D. J. (2014). Public attitudes toward immigration. Annual Review of Political Science, 17(1), 225–249. 10.1146/annurev-polisci-102512-194818

[bjos13124-bib-0038] Helbling, M. (2011). Why Swiss‐Germans dislike Germans: Opposition to culturally similar and highly skilled immigrants. European Societies, 13(1), 5–27. 10.1080/14616696.2010.533784

[bjos13124-bib-0039] Hello, E. , Scheepers, P. , & Gijsberts, M. (2002). Education and Ethnic Prejudice in Europe: Explanations for cross‐national variances in the educational effect on ethnic prejudice. Scandinavian Journal of Educational Research, 46(1), 5–24. 10.1080/00313830120115589

[bjos13124-bib-0040] Hello, E. , Scheepers, P. , & Sleegers, P. (2006). Why the more educated are less inclined to keep ethnic distance: An empirical test of four explanations. Ethnic and Racial Studies, 29(5), 959–985. 10.1080/01419870600814015

[bjos13124-bib-0041] Henrich, J. , Heine, S. J. , & Norenzayan, A. (2010). The weirdest people in the world? Behavioral and Brain Sciences, 33(2–3), 61–83. 10.1017/s0140525x0999152x 20550733

[bjos13124-bib-0042] Hjerm, M. (2001). Education, xenophobia and nationalism: A comparative analysis. Journal of Ethnic and Migration Studies, 27(1), 37–60. 10.1080/13691830124482

[bjos13124-bib-0043] Hjerm, M. , & Nagayoshi, K. (2011). The composition of the minority population as a threat: Can real economic and cultural threats explain xenophobia? International Sociology, 26(6), 815–843. 10.1177/0268580910394004

[bjos13124-bib-0044] INE‐Instituto Nacional de Estadísticas de Chile [National Institute of Statistics of Chile] . (2023). Retrieved February 7, 2024, from https://www.ine.gob.cl/estadisticas/sociales/demografia-y-vitales/demografia-y-migracion

[bjos13124-bib-0045] International Organization for Migration. (IOM) . (2017). MigFacts: International migration. Retrieved February 8, 2024, from https://reliefweb.int/report/world/migfacts‐international‐migration‐10‐jul‐2017

[bjos13124-bib-0046] Ivarsflaten, E. (2005). Threatened by diversity: Why restrictive asylum and immigration policies appeal to western Europeans. Journal of Elections, Public Opinion, and Parties, 15(1), 21–45. 10.1080/13689880500064577

[bjos13124-bib-0047] Jeannet, A. M. (2018). Revisiting the labor market competition hypothesis in a comparative perspective: Does retirement affect opinion about immigration? Research & Politics, 5(3), 1–8. 10.1177/2053168018784503

[bjos13124-bib-0048] Jenssen, A. T. , & Engesbak, H. (1994). The many faces of education: Why are people with lower education more hostile towards immigrants than people with higher education? Scandinavian Journal of Educational Research, 38(1), 33–50. 10.1080/0031383940380103

[bjos13124-bib-0049] Kalmijn, M. , & Kraaykamp, G. (2007). Social stratification and attitudes: A comparative analysis of the effects of class and education in Europe. British Journal of Sociology, 58(4), 547–576. 10.1111/j.1468-4446.2007.00166.x 18076386

[bjos13124-bib-0050] Krosnick, J. A. , & Alwin, D. F. (1989). Aging and susceptibility to attitude change. Journal of Personality and Social Psychology, 57(3), 416–425. 10.1037//0022-3514.57.3.416 2778632

[bjos13124-bib-0051] Kunst, S. C. (2022). Learning to love immigration? Testing the socialisation effect of educational field of study on lowering anti‐immigration sentiment in the Netherlands. In The educational divide in openness towards globalisation in Western Europe (pp. 5–270) (PhD thesis). University of Amsterdam.

[bjos13124-bib-0052] Lancee, B. , & Sarrasin, O. (2015). Educated preferences or selection effects? A longitudinal analysis of the impact of educational attainment on attitudes towards immigrants. European Sociological Review, 31(4), 490–501. 10.1093/esr/jcv008

[bjos13124-bib-0053] Lawrence, D. (2011). Immigration attitudes in Latin America: Culture, economics, and the Catholic Church. Latinamericanist, 55(4), 143–170. 10.1353/tla.2011.a706536

[bjos13124-bib-0054] Lawrence, D. (2015). Crossing the cordillera: Immigrant attributes and Chilean attitudes. Latin American Research Review, 50(4), 154–177. 10.1353/lar.2015.0058

[bjos13124-bib-0055] Lucassen, G. , & Lubbers, M. (2012). Who fears what? Explaining far‐right‐wing preference in Europe by distinguishing perceived cultural and economic ethnic threats. Comparative Political Studies, 45(5), 547–574. 10.1177/0010414011427851

[bjos13124-bib-0056] Maldonado, L. , & Prieto, J. (2015). Determinantes de la dinámica de la pobreza en Chile y el rol de la persistencia temporal: análisis de la Encuesta Panel Casen 2006–2009 con métodos de historia de eventos [Determinants of poverty dynamics in Chile and the role of temporal persistence: Análisis de la Encuesta Panel Casen 2006–2009 con métodos de historia de eventos]. Economia Politica, 2(2), 5–39. 10.15691/07194714.2015.005

[bjos13124-bib-0057] Manevska, K. , & Achterberg, P. (2013). Immigration and perceived ethnic threat: Cultural capital and economic explanations. European Sociological Review, 29(3), 437–449. 10.1093/esr/jcr085

[bjos13124-bib-0058] Marquart‐Pyatt, S. , & Paxton, P. (2007). In principle and in practice: Learning political tolerance in Eastern and Western Europe. Political Behavior, 29(1), 89–113. 10.1007/s11109-006-9017-2

[bjos13124-bib-0059] Matthews, R. A. , Pineault, L. , & Hong, Y. H. (2022). Normalizing the use of single‐item measures: Validation of the single‐item compendium for organizational psychology. Journal of Business and Psychology, 37(4), 639–673. 10.1007/s10869-022-09813-3

[bjos13124-bib-0060] McClosky, H. , & Brill, A. (1983). Dimensions of tolerance: What Americans believe about civil liberties. Russell Sage Foundation.

[bjos13124-bib-0061] Meseguer, C. , & Kemmerling, A. (2018). What do you fear? Anti‐immigrant sentiment in Latin America. International Migration Review, 52(1), 236–272. 10.1111/imre.12269

[bjos13124-bib-0062] Nunn, C. Z. , Crockett, H. J. , & Williams, J. A. (1978). Tolerance for nonconformity. Jossey‐Bass Publishers.

[bjos13124-bib-0063] OECD . (2022, September 19). Income inequality (indicator). 10.1787/459aa7f1-en

[bjos13124-bib-0064] Ohlander, J. , Batalova, J. , & Treas, J. (2005). Explaining educational influences on attitudes toward homosexual relations. Social Science Research, 34(4), 781–799. 10.1016/j.ssresearch.2004.12.004

[bjos13124-bib-0065] Phelan, J. , Link, B. G. , Moore, R. E. , & Stueve, A. (1995). The stigma of homelessness: The impact of the label “homeless” on attitudes toward poor persons. Social Psychology Quarterly, 60(4), 323–337. 10.2307/2787093

[bjos13124-bib-0066] Pottie‐Sherman, Y. , & Wilkes, R. (2017). Does size really matter? On the relationship between immigrant group size and anti‐immigrant prejudice. International Migration Review, 51(1), 218–250. 10.1111/imre.12191

[bjos13124-bib-0067] Runciman, W. G. (1972). Relative deprivation and social justice. A study of attitudes to social inequality in twentieth‐century England. Penguin Books.

[bjos13124-bib-0068] Schneider, S. L. (2008). Anti‐immigrant attitudes in Europe: Outgroup size and perceived ethnic threat. European Sociological Review, 24(1), 53–67. 10.1093/esr/jcm034

[bjos13124-bib-0069] Scott, R. (2022). Does university make you more liberal? Estimating the within‐individual effects of higher education on political values. Electoral Studies, 77, 1–10. 10.1016/j.electstud.2022.102471

[bjos13124-bib-0070] Selznick, G. J. , & Steinberg, S. (1979). The tenacity of prejudice: Anti‐semitism in contemporary America. Greenwood Press.

[bjos13124-bib-0071] Sirlopú, D. , & Van Oudenhoven, J. P. (2013). Is multiculturalism a viable path in Chile? Intergroup and acculturative perspectives on Chilean society and Peruvian immigrants. International Journal of Intercultural Relations, 37(6), 739–749. 10.1016/j.ijintrel.2013.09.011

[bjos13124-bib-0072] Stubager, R. (2008). Education effects on authoritarian‐libertarian values: A question of socialization. British Journal of Sociology, 59(2), 327–350. 10.1111/j.1468-4446.2008.00196.x 18498598

[bjos13124-bib-0073] Surridge, P. (2016). Education and liberalism: Pursuing the link. Oxford Review of Education, 42(2), 146–164. 10.1080/03054985.2016.1151408

[bjos13124-bib-0074] Theiler, T. (2004). The origins of Euroscepticism in German‐speaking Switzerland. European Journal of Political Research, 43(4), 635–656. 10.1111/j.1475-6765.2004.00168.x

[bjos13124-bib-0075] Uhlmann, E. , Dasgupta, N. , Elguela, A. , Greenwald, A. G. , & Swanson, J. (2002). Subgroup prejudice based on skin color among Hispanics in the United States and Latin America. Social Cognition, 20(3), 198–226. 10.1521/soco.20.3.198.21104

[bjos13124-bib-0076] Velásquez, P. , & Eger, M. A. (2022). Does higher education have liberalizing or inoculating effects? A panel study of anti‐immigrant sentiment before, during, and after the European migration crisis. European Sociological Review, 38(4), 605–628. 10.1093/esr/jcab062

[bjos13124-bib-0077] Vogt, P. W. (1997). Tolerance & education: Learning to live with diversity and difference. Sage Publications, Inc.

[bjos13124-bib-0078] Wagner, U. , & Zick, A. (1995). The relation of formal education to ethnic prejudice: Its reliability, validity and explanation. European Journal of Social Psychology, 25(1), 41–56. 10.1002/ejsp.2420250105

[bjos13124-bib-0079] Weber, H. (2022). The educational divide over feelings about ethnic minorities: Does more education really lead to less prejudice? Journal of Ethnic and Migration Studies, 48(1), 228–247. 10.1080/1369183x.2020.1810540

[bjos13124-bib-0080] Weil, F. D. (1985). The variable effects of education on liberal attitudes: A comparative‐historical analysis of anti‐semitism using public opinion survey data. American Sociological Review, 50(4), 458–474. 10.2307/2095433

[bjos13124-bib-0081] Weil, F. D. (1989). The sources and structure of legitimation in western democracies: A consolidated model tested with time‐series data in six countries since world war II. American Sociological Review, 54(5), 682. 10.2307/2117748

[bjos13124-bib-0082] World Bank . (2022). Retrieved July 27, 2022, from https://data.worldbank.org/country/chile

[bjos13124-bib-0083] Zárate, M. A. , Garcia, B. , Garza, A. A. , & Hitlan, R. T. (2004). Cultural threat and perceived realistic group conflict as dual predictors of prejudice. Journal of Experimental Social Psychology, 40(1), 99–105. 10.1016/s0022-1031(03)00067-2

