# Supplementary material for: Does educational attainment matter for attitudes toward immigrants in Chile? Assessing the causality and generalizability of higher education's so‐called “liberalizing effect” on economic and cultural threat
Source: Br J Sociol. 2024 Jun 21;75(5):731–52. doi: 10.1111/1468-4446.13124 (PMC11617814; doi:10.1111/1468-4446.13124)
Supplement: Supplementary file 1 — Supplementary Material [file BJOS-75-731-s001.docx]

Online Appendix: Does educational attainment matter for attitudes toward immigrants in Chile? Assessing the causality and generalizability of higher education’s so-called “liberalizing effect” on economic and cultural threat

Table of Contents

[**1.** **Perceived economic and cultural threat by ethnic group over time – Figure A1** 3](#_Toc167363734)

[**2.** **Perceived economic threat by ethnic group and respondents’ educational level over time – Figures A2 and A4** 4](#_Toc167363735)

[**3.** **Perceived cultural threat by ethnic group and respondents’ educational level over time – Figures A5 and A7** 6](#_Toc167363736)

[**4.** **Income as categorical and continuous variables – Figures A8-A9** 8](#_Toc167363737)

[**5.** **Age of respondents in educational transitions (%) 30 or younger and 31 or older – Table A1** 9](#_Toc167363738)

[**6.** **Educational transitions by number of observations and year/wave – Tables A2-A5** 9](#_Toc167363739)

[**7.** **Multilevel repeated measurement models with index as dependent variable – Table A6** 10](#_Toc167363740)

[**8.** **Fixed-effects models using index as dependent variable – Table A7** 11](#_Toc167363741)

[**9.** **Multilevel repeated measurement models with 4-category education variable (perceived economic threat) – Table A8** 12](#_Toc167363742)

[**10.** **Multilevel repeated measurement models with 4-category education variable (perceived economic threat) – Table A9** 13](#_Toc167363743)

# **Perceived economic and cultural threat by ethnic group over time – Figure A1**

**Figure A1.** Average perceived economic threat and cultural threat by ethnic group, over time.

# **Perceived economic threat by ethnic group and respondents’ educational level over time – Figures A2 and A4**

**Figure A2.** Perceived economic threat [Peruvians] by educational level, over time.

**Figure A3.** Perceived economic threat [Haitians] by educational level, over time.

**Figure A4.** Perceived economic threat [Venezuelans] by educational level, over time.

# **Perceived cultural threat by ethnic group and respondents’ educational level over time – Figures A5 and A7**

**Figure A5.** Perceived cultural threat [Peruvians] by educational level, over time.

**Figure A6.** Perceived cultural threat [Haitians] by educational level, over time.

**Figure A7.** Perceived cultural threat [Venezuelans] by educational level, over time.

# **Income as categorical and continuous variables – Figures A8-A9**

**Figure A8.** Monthly household income, 9 categories.


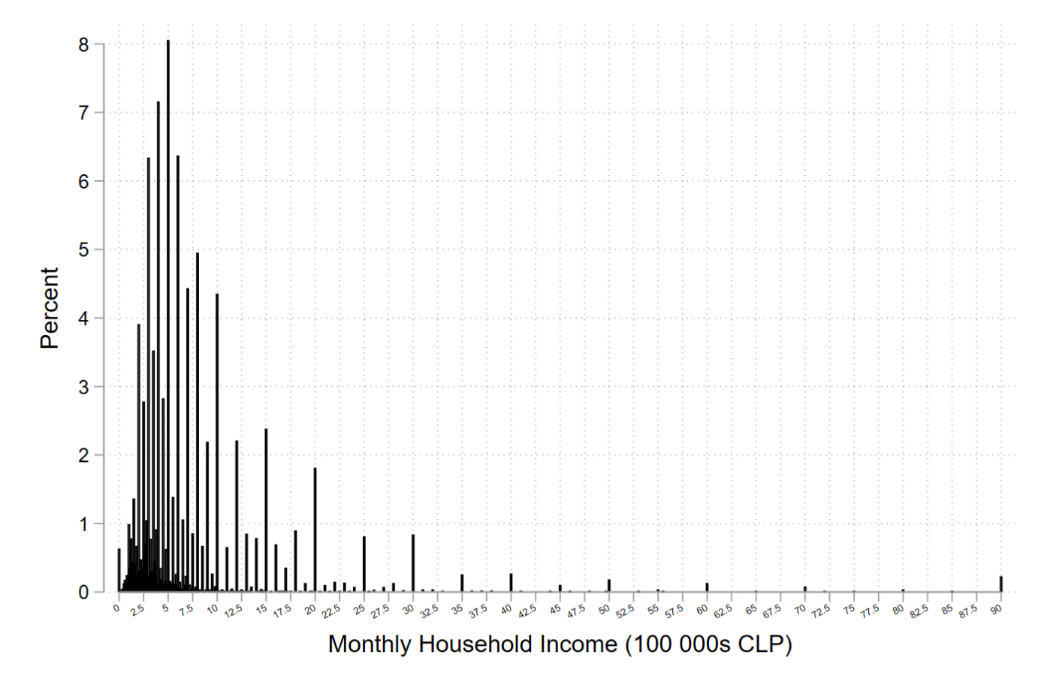


**Figure A9.** Monthly household income, continuous.

# **Age of respondents in educational transitions (%) 30 or younger and 31 or older – Table A1**

# **Educational transitions by number of observations and year/wave – Tables A2-A5**

# **Multilevel repeated measurement models with index as dependent variable – Table A6**

# **Fixed-effects models using index as dependent variable – Table A7**

# **Multilevel repeated measurement models with 4-category education variable (perceived economic threat) – Table A8**

# **Multilevel repeated measurement models with 4-category education variable (perceived cultural threat) – Table A9**
